# Evaluation of Emotions from Brain Signals on 3D VAD Space via Artificial Intelligence Techniques

**DOI:** 10.3390/diagnostics13132141

**Published:** 2023-06-22

**Authors:** Ümran Işık, Ayşegül Güven, Turgay Batbat

**Affiliations:** 1 Biomedical Engineering Graduate Program, Graduate School of Natural and Applied Sciences, Erciyes University, 38039 Kayseri, Türkiye; 2 Department of Biomedical Engineering, Engineering Faculty, Erciyes University, 38039 Kayseri, Türkiye; aguven@erciyes.edu.tr (A.G.); turgaybatbat@erciyes.edu.tr (T.B.)

**Keywords:** emotion evaluation, VAD space, artificial intelligence, EEG, SMOTE

## Abstract

Recent achievements have made emotion studies a rising field contributing to many areas, such as health technologies, brain–computer interfaces, psychology, etc. Emotional states can be evaluated in valence, arousal, and dominance (VAD) domains. Most of the work uses only VA due to the easiness of differentiation; however, very few studies use VAD like this study. Similarly, segment comparisons of emotion analysis with handcrafted features also use VA space. At this point, we primarily focused on VAD space to evaluate emotions and segmentations. The DEAP dataset is used in this study. A comprehensive analytical approach is implemented with two sub-studies: first, segmentation (Segments I–VIII), and second, binary cross-comparisons and evaluations of eight emotional states, in addition to comparisons of selected segments (III, IV, and V), class separation levels (5, 4–6, and 3–7), and unbalanced and balanced data with SMOTE. In both sub-studies, Wavelet Transform is applied to electroencephalography signals to separate the brain waves into their bands (α, β, γ, and θ bands), twenty-four attributes are extracted, and Sequential Minimum Optimization, K-Nearest Neighbors, Fuzzy Unordered Rule Induction Algorithm, Random Forest, Optimized Forest, Bagging, Random Committee, and Random Subspace are used for classification. In our study, we have obtained high accuracy results, which can be seen in the figures in the second part. The best accuracy result in this study for unbalanced data is obtained for Low Arousal–Low Valence–High Dominance and High Arousal–High Valence–Low Dominance emotion comparisons (Segment III and 4.5–5.5 class separation), and an accuracy rate of 98.94% is obtained with the IBk classifier. Data-balanced results mostly seem to outperform unbalanced results.

## 1. Introduction

Emotions emerge in our brains due to interactions caused by neural and hormonal systems [1]. If we look at emotions and their interactions in the brain in detail with examples, the brain controls emotions. The limbic system is important for emotion. The amygdala has a significant role, especially in influencing anger and aggression. Fear can appear due to the stimulated amygdala, i.e., with frightening memory flashbacks. The amygdala can enhance emotional stimuli perception. The septum influences anger and fear [2]. A phobia is an anxiety disorder occurring with extreme fear of the stimuli, such as a fear of heights (acrophobia), spiders (arachnophobia), crowds, etc. Physical and emotional symptoms appear with a phobia crisis, such as increased heart rate and breathing, sweating, and difficulty controlling emotions [3]. Besides its other vital functions, the hypothalamus is also crucial in consciousness control, regulating emotions, and responses to stress, as well as pleasure and pain [2]. Emotions affect perception, memory, creativity, behavior, etc. [1]. Perceiving and expressing emotions are abilities related to emotional intelligence. Problem-solving abilities can be positively affected by emotions. Autism also causes some difficulties in language and emotion. The internal context also affects memory retrieval of emotions, moods, consciousness, etc. [2]. Brain waves are separated into five bands: delta, theta, alpha, beta, and gamma. During a relaxed state, alpha waves dominate the cerebral cortex, gradually disappearing as emotional activity strengthens. During stressed conditions, alpha wave frequency increases and amplitude decreases, and alpha waves gradually turn to beta waves. Beta wave occurrence indicates an excited state in the cerebral cortex [4]. Chikara et al. [5] have used the fMRI–EEG system to implement a modified stop-signal task in a virtual battlefield scenario to study the neural mechanism of response inhibition under reward and punishment, which can modulate motivation and emotions and affect cognitive processing. According to their results, positive and negative monetary feedback made a difference for both behavioral indices of inhibition and brain activities.

Emotion analysis determines, compares, and evaluates emerging emotions, activating changes using various methods. Recently, emotion analysis has become increasingly attractive, and many intelligent applications for emotion recognition have been presented [6]. Artificial intelligence techniques allow computer programs to gain abilities, such as learning, solving problems, making decisions, etc. [7], and these intelligence techniques can also be used for emotion recognition despite some difficulties and limitations due to some factors, such as hiding feelings [8]. Even though emotions can be recognized by facial expressions, voice, or gesture recognition [6,9,10], these methods can be manipulated, preventing actual emotional states from being understood easily and clearly [9,10]. Because inner systems are affected less by human subjective consciousness, physiological signals can reflect emotions more truly [6]. EEG physiological brain signals remain neutral in this context and help to accurately analyze emotional states with more reliable data [9,10].

**Figure 1:**
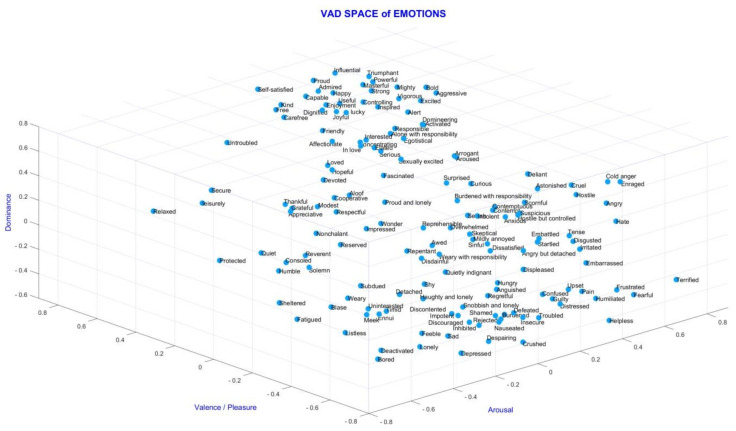
VAD space of emotions with class placements (VAD space is drawn by using the data in [14] and by taking the pleasure plane as valence).

Visual, auditory, both visual and auditory, or memory-based stimuli can be used to obtain emotions. If we look at some definitions and some studies within the scope of mood analysis, two models (discrete and dimensional) are used in emotion prediction. The discrete model has six emotions, which are anger, fear, happiness, hatred, sadness, and surprise [11]. The dimensional emotion model includes 2D and 3D models. The 2D VA model has valence and arousal dimensions. The 3D VAD model has valence, arousal, and dominance dimensions [12]. The valence dimension ranges from negative emotions, such as unpleasant/unhappy/sad, to positive emotions, such as pleasant/happy/joyful. The arousal dimension ranges from calm/bored to stimulated/excited. The dominance dimension ranges from submissive/without control to dominant/in control and empowered [12,13]. The dimensional emotion model is preferred due to its international identification and acceptance [11]. The 3D Emotion Space Model can be seen in Figure 1, which shows a three-dimensional (valence, arousal, and dominance) space that occurs using the data in [14], taking the pleasure plane as valence. Verma and Tiwary [12] proposed a 3D emotion model on VAD space for describing various emotions, including fun, joy, cheerfulness, melancholy, love, mellow, shock, etc. In general, 3D space is more sufficient and accurate for representing all emotions than 2D space.

Many studies on emotion analysis appear in the literature; however, these studies generally focus on 2D valence and arousal space. Little work focuses on 3D valence, arousal, and dominance space. In the present study, we especially wanted to study 3D space to classify emotions and to compare emotions, segmentations, and classes. EEG is used in this study to conduct space classifications and comparisons. Verma and Tiwary [12] obtained a classification accuracy (%) of 63.47 (valence), 69.62 (arousal), and 63.57 (dominance) for a tree class classification (low, medium, and high) with the DEAP database besides their own experiment. Guendil et al. [15] used a feature-based wavelet and R-ELM (extreme learning machine) classifier (with physiological signals in the DEAP database). They obtained classification rates of 73.43% (V), 72.65% (A), and 69.3% (D) for the two classes. They obtained the best average classification accuracy of 53% for four class classifications in the VA quadrant. Özel et al. [16] studied multivariate synchro squeezing transform on EEG signals from the DEAP database for feature extraction to classify emotions. They used a 2D VA model for four emotional states and obtained the best accuracy of 79.1%. They used a 3D VAD model for eight emotional states with an SVM classifier and obtained the highest accuracy of 93%. Accuracies are higher for eight emotions than in the four emotion model.

The classification and comparison of emotions could contribute to many areas, such as intelligent health technologies, brain–computer interfaces (BCIs), psychology, patient robots, humanoid robot technologies, virtual reality, holograms, simulation systems, game technologies, interactive education systems, smart homes, workplaces, driver systems, etc. This study contributes to the literature with its comparisons and comprehensive analytical approach implemented with two sub-studies: first, a segmentation pre-study, and second, an emotion comparison and evaluation study, including comparisons of selected segments, class separation levels, and data balancing.

Our study’s general results show that accuracies obtained from balanced datasets mostly seem to outperform those obtained from unbalanced datasets. The accuracies of unbalanced data in the figures of the second part of this study generally seem to increase in the order of A&G, D&F, B&H, and C&E comparisons. We have obtained high accuracy results that can be seen directly in the spider web diagrams in the second part of this study. The best accuracy result in this study for unbalanced data is for the C&E comparison (Seg III and 4.5–5.5 class separation), and an accuracy rate of 98.94% CCI (F1: 98.9% and MCC: 97.9%) is obtained with the IBk classifier, which is greater than the compared studies from the literature which has a dominance dimension besides valence and arousal.

This study is divided into two sub-studies. In the first part of this study, the comparison of the VAD emotions via various segmentations of EEG signals is objected to. Segments I–VIII are obtained by applying the non-overlapping windowing method. In the second part of this study, Segments III, IV, and V are chosen, and the two class cross-comparisons of the VAD emotions, segments, and class levels are objected to. In both parts of this study, Discrete Wavelet Transform (DWT) is applied to EEG signals to separate the brain waves into their bands; d_1_, d_2_, d_3_, and a_3_ wavelet coefficients are obtained, which correspond to gamma, beta, alpha, and theta bands (γ, β, α, and θ bands), respectively. Attributes are also calculated for all channels for each of the 32 participants and for each of the 40 video pieces. After obtaining the feature datasets, classifications are conducted with classifiers, such as SMO, FURIA, etc. Because of the unbalanced classes, data balancing and comparing balanced and unbalanced results are also targeted in this study. In this context, the SMOTE method is used to pre-filter the dataset for oversampling of the minority classes. Classifications are conducted both with and without the SMOTE method in this study. This article is organized into five sections. Section 2 describes the materials and methods used in this study in detail with its subsections (Section 2.1. Dataset and Channels; Section 2.2. Overall Method and Classification; Section 2.3. Classes; Section 2.4. Attributes; Section 2.5. SMOTE; Section 2.6. Classifiers; and Section 2.7. Metrics). Section 3 presents the results for the two sub-studies (Section 3.1. Segmentation Pre-Study and Section 3.2. Cross-Comparisons of 3D VAD Emotions, Segments, and Classes). Section 4 includes a discussion of the results. Section 5 consists of the conclusion.

## 2. Materials and Methods

### 2.1. Dataset and Channels

**Figure 2:**
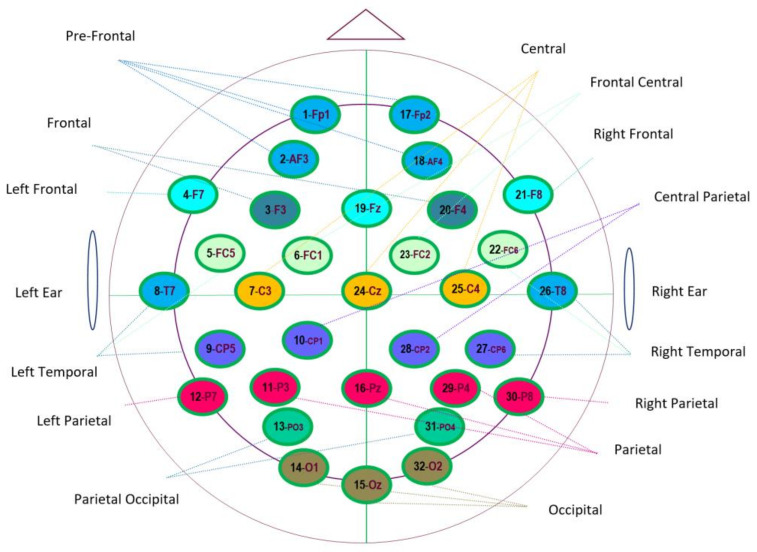
32 Channel placement of EEG according to the 10–20 electrode placement system (numbers indicate the channel numbers of EEG signals from the DEAP dataset).

The DEAP database is used in this study. It is a multimodal database for emotion analysis. The EEG and peripheral physiological signals of 32 participants (16 males and 16 females) aged between 19 and 37 (mean 26.9) and facial videos of 22 of the participants were recorded while they were watching 40 music videos, each of which was 1 min long. Self-assessments were also performed by the participants in addition to signal recordings. The participants rated each video according to their arousal, valence, liking, dominance, and familiarity levels (with continuous 1–9 scaled self-assessment manikins, except for familiarity, rated with a discrete 1–5 scaled manikin). The database contains both preprocessed and unprocessed data. EEG data were down-sampled to 128 Hz, EOG artifacts were removed, and a band-pass frequency filter of 4.0–45.0 Hz was applied. The data were also down-sampled to 128 Hz as a preprocessing measure for the other channels (EOG, EMG, GS, respiratory belt, plethysmography, and temperature) [17]. In the present study, preprocessed DEAP data were used. The 32 EEG channels used in this study are given in Figure 2.

### 2.2. Overall Method and Classification

**Figure 3:**
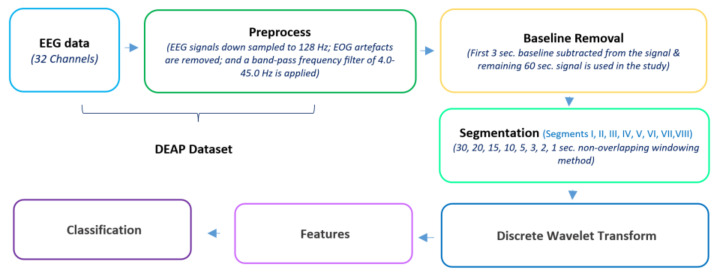
Flow chart for EEG signals analysis.

The first three seconds of the EEG data from the DEAP dataset are subtracted from the signal, and the remaining one minute signal is used for further operations in this study. The windowing method is used for the segmentation of 60 s EEG signals. The flow chart for EEG signal analysis is given in Figure 3.

**Figure 4:**
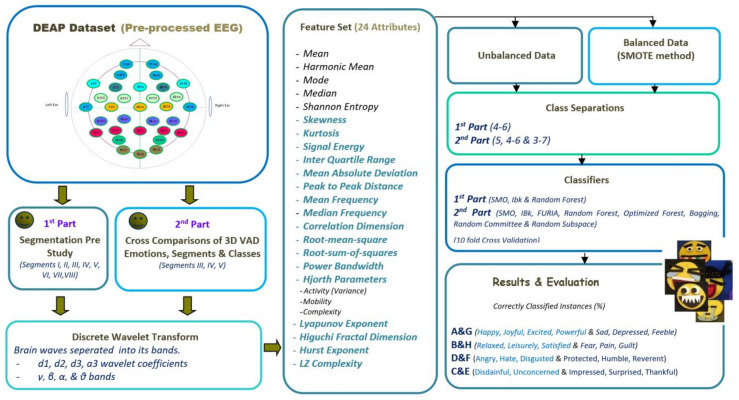
Flow chart of the overall method.

This study is divided into two parts. The flow chart of the overall method is given in Figure 4. The segmentation of EEG signals is studied in the first part. The 30, 20, 15, 10, 5, 3, 2, and 1 s non-overlapping windowing method is used to segment the signals. Here, time in seconds refers to the fact that the 60 s signal will be divided into segments. Windowing separations are titled with segment names, and the 2, 3, 4, 6, 12, 20, 30, and 60 signal pieces correspond to 30, 20, 15, 10, 5, 3, 2, and 1 s windowing, and these correspond to Segments I, II, III, IV, V, VI, VII, and VIII, respectively. For instance, Segment I means that the 60 s EEG signal is divided into 2 segments with 30 s windowing. N means no segmentation is applied to the signal (Section 3.1. Segmentation Pre-Study).

In the second part of this study, Segments III, IV, and V, which correspond to 15, 10, and 5 s windowing, are chosen for the 2 class cross-comparisons of the VAD areas (3.2. Cross-Comparisons of 3D VAD emotions, segments, and classes).

Wavelet Transform is a powerful statistical tool applied to various problems, and it has proven its effectiveness in various areas, such as time–frequency analysis of signals, image processing, denoising and extraction of weak signals, data compression, medical image technology, pattern recognition, speech recognition, fingerprint verification, etc. [18,19]. The local analysis of a signal is performed using the wavelets in this method. Unlike the Fourier Transform, which uses the time–frequency domain and constant window function, Wavelet Transform uses the time–scale domain and continuously varying window function [20]. Different mother wavelets, such as Harr, Daubechies, etc., can be used in Discrete Wavelet Transform (DWT) [18]. In both parts of this study, DWT is applied to the complete EEG signal as well as to all segments of the signal, and d1, d2, d3, and a3 wavelet coefficients are obtained. A Reverse Bior wavelet is used for the analysis.

To form the feature datasets for each signal or segment, the mean, Shannon entropy, variance, harmonic mean, mode, skewness, kurtosis, signal energy, Higuchi fractal dimension, mobility, complexity, LZ complexity, Lyapunov exponent, Hurst exponent, median, peak to peak distance, root mean square, root sum of squares, mean frequency, median frequency, power bandwidth, correlation dimension, mean absolute deviation, and interquartile range attributes are used in this study (24 attributes). They are calculated for all channels for each of the 32 participants and for each of the 40 video pieces.

After obtaining the feature datasets, Random Forest, SMO, IBk (1-Manh), FURIA, Bagging, Random Committee, and Random Subspace classifiers are studied and the results, such as accuracy rates (CCI %, F1 scores, and MCC values), are obtained for the data. In all classifications, 10-fold cross-validation is applied to the data.

A SMOTE filter is also applied to the data to balance the classes. The oversampling SMOTE method is applied, and synthetic samples are generated for the minority class. All minority classes are converged to the majority class for data balancing. Both filtered and unfiltered results are obtained for the comparison of emotions.

We generally compared eight three-dimensional emotion regions in this study: A: happy, joyful, useful, powerful, influential, friendly, excited, and inspired; B: relaxed, leisurely, untroubled, and satisfied; C: disdainful and unconcerned; D: angry, hostile, cruel, insolent, hate, and disgusted; E: impressed, surprised, thankful, and curious; F: protected, humble, and reverent. These emotion groups we compared are discussed in the discussion section in more detail. Besides the discussion section, cross-comparisons can also be seen in Figure 4.

The MATLAB program (R2022a) calculates the wavelets, 32 EEG channels, and attributes, and the WEKA program (Waikato Environment for Knowledge Analysis) version 3.9.6. is used for the classification [21].

### 2.3. Classes

**Figure 5:**
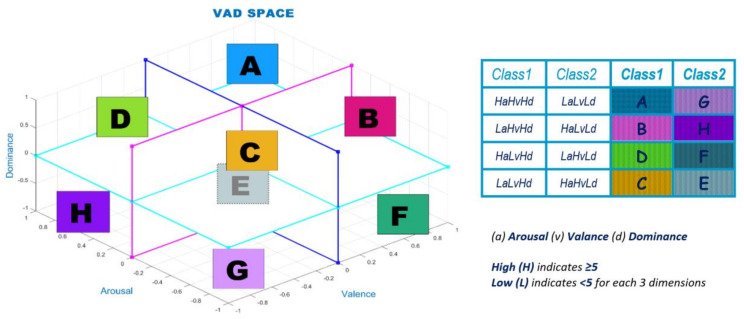
Class placements on VAD space (here, a 0-midpoint on VAD space indicates 5-midpoint for the 1–9 scale, and values below and above this scale also indicate the same logical separation related to the midpoint).

In this study, classes are set by considering high and low values of valence, arousal, and dominance scores. Class placements on VAD space are shown in Figure 5, in which the emotions are placed in the eight emotion regions. The valence dimension is considered high and low (not as positive or negative), just like the other dimensions (valence and dominance) in this study, to simplify the understanding.

The letters in Figure 5 indicate the combined coordinates of arousal, valence, and dominance, respectively: A (HaHvHd); B (LaHvHd); C (LaLvHd); D (HaLvHd); E (HaHvLd); F (LaHvLd); G (LaLvLd); and H (HaLvLd). Four comparisons are conducted between the 3-dimensional regions as A&G (HaHvHd-LaLvLd), B&H (LaHvHd-HaLvLd), D&F (HaLvHd-LaHvLd), and C&E (LaLvHd-HaHvLd). In total, 5, 4–6, and 3–7 class separations are used for the A&G, B&H, and D&F emotion comparisons. Furthermore, 5, 4.5–5.5, and 4–6 class separations are used for the C&E emotion comparisons. 0-midpoint on VAD space in Figure 5 indicates 5-midpoint for the 1–9 scale for 5 class separation. For instance, for a 4–6 separation, a high value refers to ≥6, and a low value refers to ≤4.

### 2.4. Attributes

Brief definitions of the attributes used are given below.

The sample mean is the numerical average of the data. Variance can be defined as the average of the square of the differences from the mean [22]. The harmonic mean is defined as the reciprocal of the arithmetic mean of the reciprocals of the values in the observation, while the mode is the value that occurs most frequently, and the median is the value of the variable that divides the distribution into two parts [23]. Shannon entropy is proposed to measure how the signal information can be quantified with absolute precision [24]. Skewness is a 3rd order moment, which shows asymmetry of the data distribution about the mean, while kurtosis is a 4th order moment indicating the major peaks in the signal’s time domain [25]. If s(t) is a signal, the signal power is s”(t), and the signal energy is [26]

(1) $$E=\int_{-\infty }^{\infty }s^{2}\left(t\right)\mathrm{dt}$$

The interquartile range (IQR) is a statistical distribution measure, and it is defined as the difference between the 3rd–1st quartiles (Q3–Q1) [27]. The mean absolute deviation (MAD) is defined as the average of absolute distances from the data mean. For n number of observations (x_1_, x_2_…, x_n_)

(2) $$\mathrm{MAD}=\frac{\sum_{k=1}^{n}⃒x_{i}-{\overset{-}{x}}_{i}\;⃒}{n}$$

where $\overset{-}{x}$ is the mean of the data [28]. The mean frequency (MNF) is defined as the sum of the product of the signal power spectrum and the frequency divided by the total sum of the power spectrum.

(3) $$\mathrm{MNF}=\sum_{j=1}^{M}f_{j}P_{j}/\sum_{j=1}^{M}P_{j}$$

Pj is the power spectrum at a frequency bin j, fj is the frequency of the spectrum at a frequency bin j, and M is the total number of frequency bins [29]. The median frequency (MDF) is a frequency at which the signal power spectrum is divided into two regions with equal integrated power [29].

(4) $$\mathrm{MDF}=\sum_{j=1}^{M}P_{j}=\sum_{j=\mathrm{MDF}}^{M}P_{j}=\frac{1}{2}\sum_{j=1}^{M}P_{j}$$

The correlation dimension (CD) is a method for determining the correlation of a time domain signal sampled uniformly. It is a method to determine the dimension of a nonlinear signal [30].

(5) $$C\left(r\right)=\frac{2}{N\left(N-1\right)}\sum_{\left(i\neq j\right)}\theta (r-|X\left(i\right)-X\left(j\right)|$$

divided into two regions with an equal integrated power.

Root mean square (RMS) is the square root of the mean square value, which is defined for a specific interval of time. RSSQ is the root sum of squares [31]. The power bandwidth (PBW) is defined as the measure of the frequency difference between the points where the spectrum falls at least 3 dB below the reference level [32].

The Hjorth parameters (HP), extracted by Hjorth in 1970, are activity, mobility, and complexity. Activity can be defined as the information of the signal power; mobility is an estimation of the mean frequency; and complexity is the bandwidth, the change in frequency, or the standard deviation of the power spectrum. HPs are commonly used in biological signals and are suitable for non-stationary EEG signal analysis [25,33].

(6) $$\mathrm{Activity}=\mathrm{Var}(y\left(t\right));$$

(7) $$\mathrm{Mobility}=\sqrt{\frac{\mathrm{Var}\left(\frac{\mathrm{dy}(t)}{\mathrm{dt}}\right)}{\mathrm{Var}\left(y(t)\right)}}$$

(8) $$\mathrm{Complexity}=\frac{\mathrm{Mobility}\left(\frac{\mathrm{dy}(t)}{\mathrm{dt}}\right)}{\mathrm{Mobility}\left(y(t)\right)}$$

The Lyapunov exponent (LE) measures the exponential convergence or divergence by considering the exponent to be lower or higher than 0, respectively [24]. lyap_r indicates the largest LE, while lyap_e indicates the whole spectrum of LE, whose positive exponents indicate chaos and unpredictability. LE measures total predictability [34]. It is a strong chaos indicator if it has a positive value, and as it increases, chaos increases while predictability decreases. The largest Lyapunov exponent (LLE) is used chiefly for the nonlinear analysis of physiological signals, and it is sufficient for chaos evaluation. Many algorithms can be used to calculate LE and LLE [30].

The Higuchi fractal dimension (HFD) method was first developed in 1988 by Higuchi et al. For a finite set of time series, if a new time series is obtained and the Lm(k) average value is calculated, this indicates the curve length (m is the initial time and k is the interval time). The HFD is then calculated in the slope between ln(L(k)) and ln(1/k) values [35].

First developed by Harold Edwin Hurst, the Hurst exponent (HE) statistically calculates the time series’ variability [24]. Considering a time series that increased previously, it is possible to identify if it is likely to increase more, less, or equally like the previous steps with the Hurst exponent [35]. The HE value can range from 0 to 1. If it is ranged as 0 < HE > 0.5, then the time series has long-ranged anti-correlations; if it is equal to 0.5, there are no correlations; if it is ranged as 0.5 < HE > 1, then the time series has long-ranged correlations; and, if the value equals to 1, then there is self-similarity in the time series [24]. Detrended fluctuation analysis (DFA) also uses a remarkably similar parameter to HE called the Hurst parameter H [35].

Lempel–Ziv (LZ) complexity is used frequently in the irregularity analysis of biomedical signals with its interpretability and suitability. LZ complexity measures the nonparametric complexity of the irregularity of time series [36]. LZ complexity evaluates the algorithmic complexity and determines the emergence rate of new patterns [37]. A greater LZ complexity means the new changes occur at a faster rate, indicating data change irregularly. In contrast, a lower LZ complexity means a slower rate of changes, and the data change regularly and periodically [36].

### 2.5. SMOTE

SMOTE is a data balancing method. If sample numbers of the classes in a dataset are not approximately equal, then there is an imbalance between the classes. If the data are imbalanced with different class sizes, using predictive accuracy would not be appropriate for classification [38]. Because there are imbalanced classes in our data from the DEAP dataset, we also used SMOTE to oversample our minority classes. Both classifications with and without the SMOTE method are performed in this study.

For the oversampling SMOTE method, synthetic samples are generated for the minority class. The sample and its nearest neighbor and the distance metric method are considered for creating a difference between them, which is multiplied by a random value at a [0, 1] interval and then added to the feature vector [39]. According to the results of Chawla et al. [38], for a minority class, SMOTE can improve accuracy, and it provides an innovative approach to oversampling. SMOTE also enhances the performance of the under-sampling of the majority class.

### 2.6. Classifiers

The classifiers used in this study are SMO from Function Classifiers, IBk from Lazy Classifiers, Random Forest and Optimized Forest from the Tree Structures, FURIA (Fuzzy Unordered Rule Induction Algorithm) from Rules, Bagging, Random Committee, and Random Subspace from Meta Classifiers. Classifiers are used with default parameters of the WEKA program, except for IBk. A 10-fold cross-validation is applied to the data during classifications.

IBk (Instance-Based Learning): the K-Nearest Neighbor (KNN) algorithm (lazy learning) is a supervised learning method used in a wide variety of fields in machine learning areas, such as pattern recognition, signal, image processing, etc. [25]. IBk is a KNN classifier. Several nearest neighbors can be explicitly specified or automatically determined. Different search algorithms can be used to speed up the finding process of nearest neighbors. IBk uses the same distance metric as KNN, and the distance function is a search method parameter [40]. The KNN classifier uses distances, such as Euclidean, Manhattan, Chebychev, Minkowski, etc., to determine the nearest neighbor in feature space [25]. This study uses Manhattan distance and 1 nearest neighbor parameters (K = 1) for classification.

SMO (Sequential Minimum Optimization): SVM is another supervised learning method [25], and an SVM quadratic programming problem can be easily solved with SMO. It is a simple algorithm for solving the problem by separating it into subproblems that are solved extremely fast, causing the overall problem to be solved too rapidly [41].

It is possible to achieve accurate classification and regression with suitable randomness. Random Forest (RF) is developed by Breiman, and it is an effective tool in prediction [42]. An ensemble classification method with high accuracy results, it randomly builds multiple trees in subspaces of the feature space [43]. The general error depends on the strength and correlation of the individual trees in the forest [42].

FURIA (Fuzzy Unordered Rule Induction Algorithm) is an advanced version of the RIPPER algorithm, which is a rule learner after some modifications and extensions. For instance, it uses fuzzy rules instead of conventional rules, it uses unordered rule sets instead of rule lists, and it uses a novel rule stretching technique. According to some experiments, FURIA outperforms the original RIPPER algorithm and some other fuzzy rule learning methods [44].

The Bagging method generates predictor versions by making bootstrap replicates of the learning set, and then it uses them to obtain an aggregated predictor. Classification and regression experiments with real and simulated datasets show that Bagging can significantly improve accuracy [45].

Random Committee (RC) is a classification method that builds an ensemble of randomizable base classifiers. Different random number seeds are used for each base classification, and predictions from each are averages to form the overall prediction value [21].

Random Subspace (RSS) is formed by multiple trees such that subsets will be selected pseudo-randomly from the feature vector. It forms a decision-tree-based classifier with the highest training data accuracy [21].

The Optimized Forest (OF) algorithm determines the number of trees for maintaining an optimal sub-forest using a Genetic-Algorithm-based technique. The method selects a small sub-forest from a large forest. An optimum number of diverse and accurate trees could improve the absolute accuracy besides its computational advantage, as many trees could be less valuable in a forest [46].

### 2.7. Metrics

The present article has provided % accuracies (Correctly Classified Instances—CCI (%)). Some other performance measures, which are F1 scores and MCC values, have also been calculated for all the results in this study. However, CCI values (%) have been used in most of the figures in this study because CCI is the most used performance metric in similar studies in the literature.

If the elements of the confusion matrix are Tp (True Positives), Tn (True Negatives), Fp (False Positives), and Fn (False Negatives), accuracy is defined as [47]:

(9) $$\mathrm{Accuracy}=\frac{\mathrm{Tp}+\mathrm{Tn}}{n^{+}+n^{-}}=\frac{\mathrm{Tp}+\mathrm{Tn}}{\mathrm{Tp}+\mathrm{Tn}+\mathrm{Fp}+\mathrm{Fn}}$$

The F1 value is the harmonic mean of precision and sensitivity. Precision (Positive Predictive Value) is the ratio of true positives to all positives. Sensitivity (recall/true positive rate) is the ratio of positives to all true positives [47,48].

(10) $$F1\;\mathrm{Score}=\frac{2.\mathrm{Tp}}{2.\mathrm{Tp}+\mathrm{Fp}+\mathrm{Fn}}=2\frac{\mathrm{Precision}.\mathrm{Sensitivity}}{\mathrm{Precision}+\mathrm{Sensitivity}}$$

Matthew’s correlation coefficient (MCC) is a method of contingency matrix that calculates the Pearson product–moment correlation coefficient between actual and predicted values [47]:

(11) $$\mathrm{MCC}=\frac{\mathrm{Tp}.\mathrm{Tn}-\mathrm{Fp}.\mathrm{Fn}}{\sqrt{\left(\mathrm{Tp}+\mathrm{Fp}\right).\left(\mathrm{Tp}+\mathrm{Fn}\right).\left(\mathrm{Tn}+\mathrm{Fp}\right).(\mathrm{Tn}+\mathrm{Fn})}}$$

The F1 score differs from MCC, and accuracy performance measures for it are independent of True Negatives; it is also not symmetric for class swapping [47].

## 3. Results

This study is divided into two parts: the segmentation pre-study and cross-comparisons of 3D VAD emotions, segments, and classes. For the comparisons of the segmentations for emotion analysis, we used all valence, arousal, and dominance coordinates together for two class cross-comparisons in both parts of this study. Class comparisons are made for the class separation levels in this study, which are given in Table 1.

### 3.1. Segmentation Pre-Study

In this section of this study, the 30, 20, 15, 10, 5, 3, 2, and 1 s non-overlapping windowing method is used for the segmentation of EEG signals corresponding to Segments I, II, III, IV, V, VI, VII, and VIII, respectively, as given in Table 2. Feature sets are generated for all segmented data, and then these pieces are assembled to form the related segments’ sub-dataset for further use.

**Figure 6:**
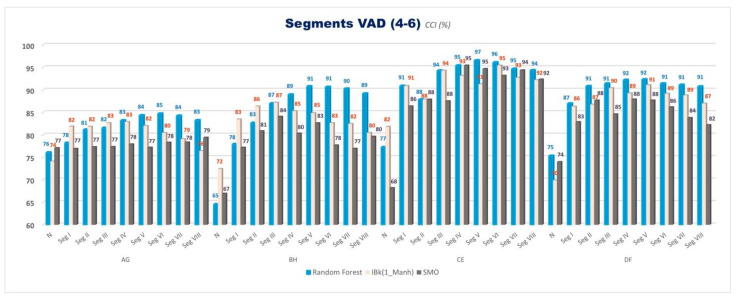
CCI (%) values for VAD space for SMO, IBk, and Random Forest Classifiers for 4–6 classes for all segments (*N*: No Segment and Segments I–VIII).

**Figure 7:**
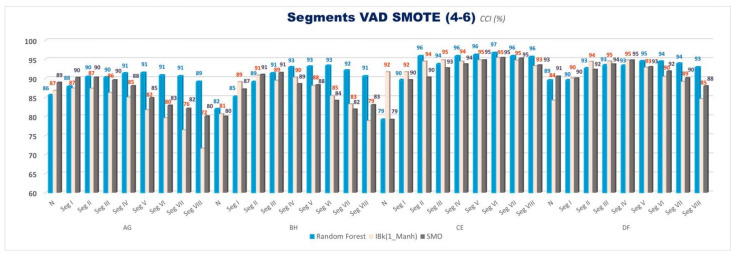
Balanced data CCI (%) values for VAD space for SMO, IBk, and Random Forest Classifiers for 4–6 classes for all segments (*N*: No Segment and Segments I–VIII).

Correctly Classified Instances (CCI %) for VAD space binary emotion comparisons for evaluating the effect of eight segments are given in Figure 6. Accuracies with the balanced data with SMOTE are given in Figure 7. Only three classifiers (SMO, IBk, and Random Forest) are used to classify in this part of this study. Similarly, this part of this study considers only 4–6 class separation levels.

### 3.2. Cross-Comparisons of 3D VAD Emotions, Segments, and Classes

In the second part of this study, Segments III, IV, and V, corresponding to 15, 10, and 5 s windowing, are used for emotion comparisons. Here, Segments III, IV, and V mean that the 60 s EEG signal is divided into 4, 6, and 12 segments/pieces with 15, 10, and 5 s windowing, respectively. N means a complete 60 s signal processed without segmentation (Table 2).

In total, 5, 4–6, and 3–7 class separation levels are used for A&G, B&H, and D&F emotion comparisons. Furthermore, 5, 4.5–5.5, and 4–6 class separation levels are used for C&E emotion comparisons (Table 1).

**Figure 8:**
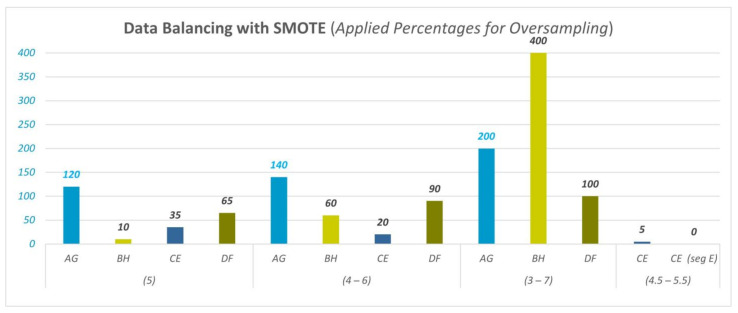
Data balancing with SMOTE (applied percentages for oversampling).

The data balancing method with a SMOTE filter is also applied to the minority classes in this part of this study. Synthetic samples are generated for the minority class with oversampling. All minority classes for the binary cross-comparisons are converged to the majority class by increasing the applied percentages 5 by 5 until reaching a closer data size to the majority class (here, the aim is to achieve close to, but not to exceed, class size). Applied percentages for oversampling for data balancing are given in Figure 8.

All class sizes are not given here. However, some examples are given: for D&F SegD 4–6 separation, high and low classes have 288 and 150 samples, respectively, and after applying data balancing with a percentage of 90%, low-class samples increase to 285; for C&E SegD 5 separation, high and low classes have 480 and 348 samples, respectively, and after applying data balancing with a percentage of 35%, low-class increases to 469 samples.

**Figure 9:**
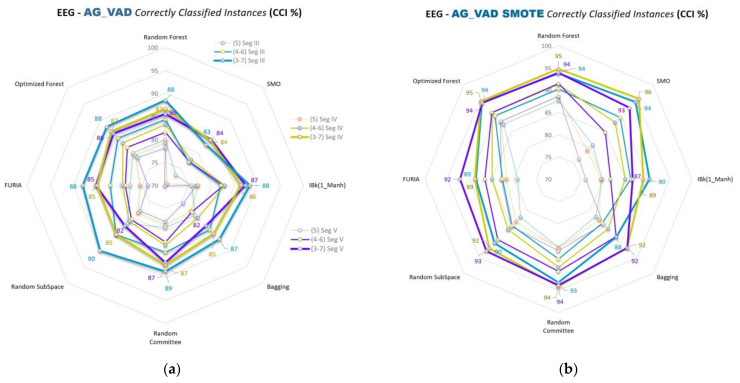
CCI (%) values for A&G comparisons of VAD space for Segments III, IV, and V for 5, 4–6, and 3–7 classifications by using: (**a**) Unbalanced data; (**b**) Balanced data.

**Figure 10:**
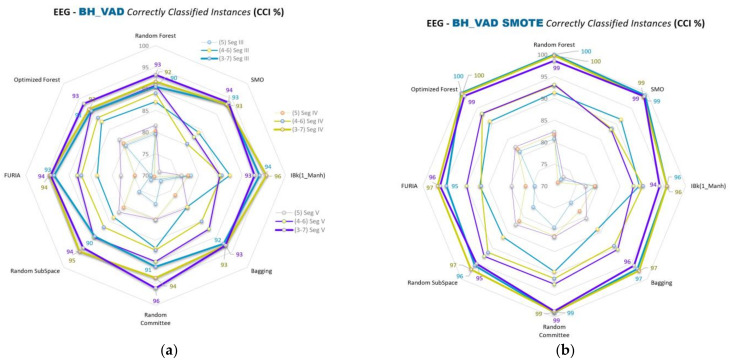
CCI (%) values for B&H comparisons of VAD space for Segments III, IV, and V for 5, 4–6, and 3–7 classifications by using: (**a**) Unbalanced data; (**b**) Balanced data.

**Figure 11:**
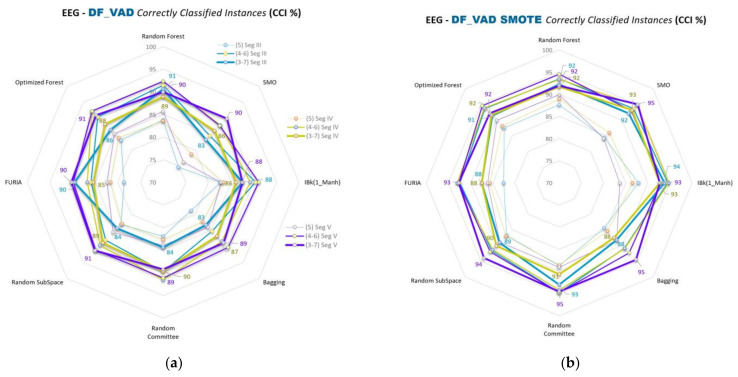
CCI (%) values for D&F comparisons of VAD space for segments III, IV, and V for 5, 4–6, and 3–7 classifications by using: (**a**) Unbalanced data; (**b**) Balanced data.

**Figure 12:**
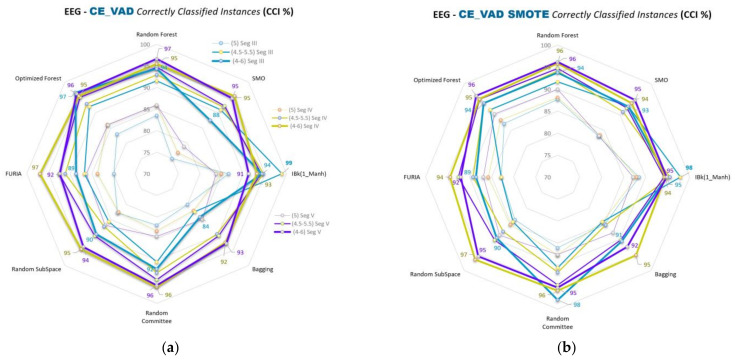
CCI (%) values for C&E comparisons of VAD space for segments III, IV, V for 5, 4.5–5.5, and 4–6 classifications by using: (**a**) Unbalanced data; (**b**) Balanced data.

Spider web graphics of VAD space emotion comparisons (A&G, B&H, D&F, and C&E) and the segment and class separation level comparisons of the emotions are given in Figure 9, Figure 10, Figure 11 and Figure 12, respectively. This part of this study uses SMO, IBk, FURIA, Random Forest, Optimized Forest, Bagging, Random Committee, and Random Subspace classifiers. The % accuracies (CCI %) obtained with all these classifiers are given in the figures below in this part of this study.

Both the filtered and unfiltered results are obtained for the comparison of emotions. All the related figures have unfiltered dataset results on the left sides, indicated with the letter, and filtered versions (balanced with SMOTE) on the right sides of the figures are indicated with the letter b.

CCI (%) values for A&G comparisons of VAD space for Segments III, IV, and V are given in Figure 9a,b for 5, 4–6, and 3–7 class separations to evaluate the emotions. Figure 9b is the SMOTE-applied version of the classification.

CCI (%) values of B&H comparisons of VAD space for Segments III, IV, and V are given in Figure 10a,b for 5, 4–6, and 3–7 class separations. Figure 10b is the SMOTE-applied version of the classification.

CCI (%) values of D&F comparisons of VAD space for Segments III, IV, and V are given in Figure 11a,b for 5, 4–6, and 3–7 class separations.

CCI (%) values for C&E comparisons of VAD space for segments III, IV, and V are given in Figure 12a,b. Here, 5, 4.5–5.5, and 4–6 class separations are used, which are different from the above comparisons in Figure 9, Figure 10 and Figure 11, which use 5, 4–6, and 3–7 class separations. The 3–7 class separation is not used here because it has so few sample sizes for minority classes for non-segmented data. As such, the 4.5–5.5 class separation level has been added only for comparisons evaluating C&E area emotions.

**Figure 13:**
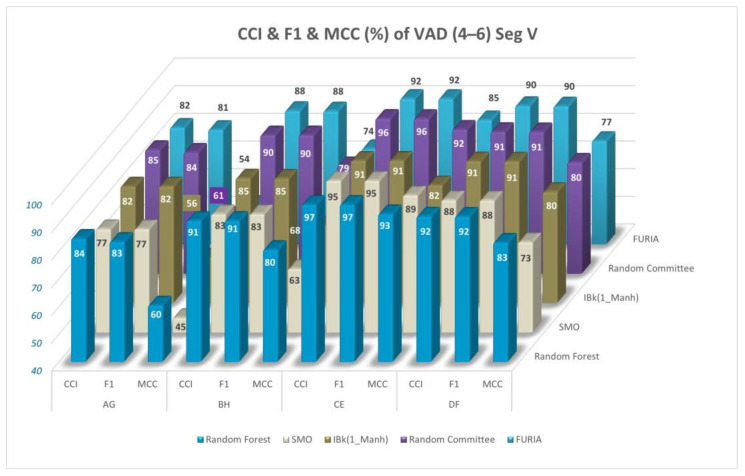
CCI, MCC values, and F1 scores (%) for comparisons of VAD space (Segment V, 4–6 classification).

CCI, MCC values, and F1 scores (%) for the comparisons of VAD space and the comparison of performance metrics are given in Figure 13. Only Segment V and the 4–6 classification are used in the figure. The results are given for the Random Forest, SMO, IBk, FURIA, and Random Committee classifiers.

**Figure 14:**
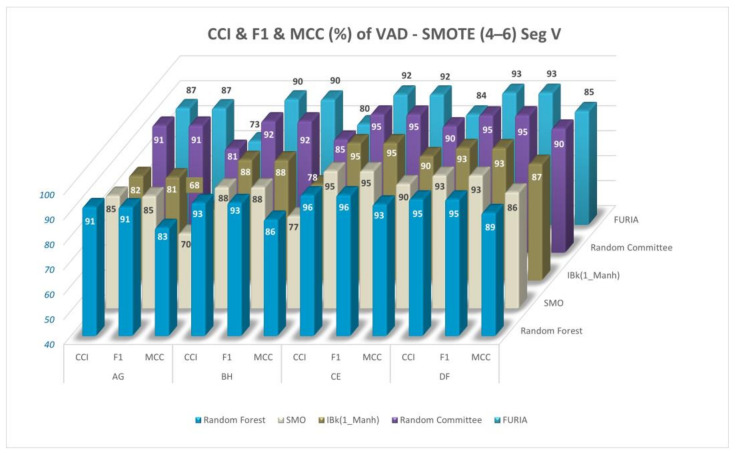
CCI, MCC values, and F1 scores (%) for the comparisons of VAD space (data balanced with SMOTE) (Segment V, 4–6 classification).

CCI, MCC values, and F1 scores (%) for the comparisons of VAD space and the comparison of performance metrics (data balanced with SMOTE) (Segment V, 4–6 classification) are given in Figure 14 for the Random Forest, SMO, IBk, FURIA, and Random Committee classifiers.

## 4. Discussion

In the literature, we generally see studies comparing emotions with binary classifications, such as high and low levels of valence and high and low levels of arousal, which divide the VA space into two pieces. Some studies also consider two-dimensional VA space, which divides the space into four pieces.

There are many studies on emotion analysis in the literature. However, these studies generally consider VA space. Little work seems to focus on the 3D space of valence, arousal, and dominance, which divides the VAD space into eight pieces weighing the dominance besides valence and arousal dimensions. Therefore, in this study, we especially wanted to focus on 3D VAD space to classify, compare, and evaluate emotions.

Similar to these comparisons of segmentations for emotion analysis using handcrafted feature sets that the researcher manually considers, we also see valence and arousal dimensions in the literature. In this context, we also wanted to focus on 3D VAD space to study the segmentations of EEG signals for emotion analysis.

There are few studies for emotion analysis using the SMOTE method. Because of the unbalanced classes for our comparisons, we also wanted to use this filter (oversampling of the minority class) to perform data balancing besides the unfiltered data.

This study is divided into two parts: a segmentation pre-study and cross-comparisons of 3D VAD emotions, segments, and classes. We used all valence, arousal, and dominance coordinates together for two class cross-comparisons in both studies. In the first part of this study, the 30, 20, 15, 10, 5, 3, 2, and 1 s non-overlapping windowing method is used for the segmentation of EEG signals, which correspond to Segments I, II, III, IV, V, VI, VII, and VIII, respectively (Table 2). The % CCI values are calculated for VAD space binary emotion comparisons (A&G, B&H, C&E, and D&F) for evaluating the segmentation effect. Eight segments (Segments I, II, III, IV, V, VI, VII, and VIII) are given in Figure 6 and Figure 7 (data-balanced version). Only three classifiers (SMO, IBk, and Random Forest) are used for the classification in this part of this study. Similarly, this part of this study considers only the 4–6 class separation level.

Ahmed et al. [49] studied a novel method (InvBase) for emotion classification after baseline removal. The results show that baseline removal gave higher accuracy with simple features, requiring less computation time. They also concluded in their study that the emotion features were dominant in a range of 1 s to 12 s time slots. Similarly to that study, we have subtracted the first three seconds of the preprocessed EEG data from the signal, and the remaining 60 s signal is used in this study.

From Figure 6, it can be seen that segmentation increases accuracy. The results show that accuracies generally grow to some extent as the segmentation increases; however, after some increase, they give similar results or even begin to decrease at the end parts for every three classifiers (SMO, IBk, and Random Forest). Segments III, IV, and V, which correspond to 15, 10, and 5 s windowing, are chosen to use in the second sub-study, which has more complex comparisons with the class separation levels, classifiers, and data balancing.

In the second part of this study, spider web diagrams compare the results for the binary cross-comparisons of 3D VAD emotions, segments, class separations, classifiers, and data balancing. Lines are drawn to ensure a more accessible and precise understanding of the results and the comparisons. Data points on the lines indicate the classifiers’ results (% accuracies). This part of this study uses SMO, IBk, FURIA, Random Forest, Optimized Forest, Bagging, Random Committee, and Random Subspace classifiers. Comparisons of segments III, IV, and V can be seen more clearly in this sub-study. Different colored lines, including data points of related classifiers in the figures, indicate the segmentations. Blue, green, and purple colored lines correspond to Segments III, IV, and V, respectively.

More opening parts in the middles of the figures mean the segment separations are closer. SMOTE versions of each figure in this part of this study show that those openings in the middle parts are increased compared to the unbalanced ones (Figure 9b, Figure 10b, Figure 11b, and Figure 12b). As such, the results of the segment classifications are closer to each other.

Class separations 5, 4–6, and 3–7 are used for the A&G, B&H, and D&F emotion comparisons. Class separations 5, 4.5–5.5, and 4–6 are used for the C&E emotion comparisons. Sizes of the same colored lines (segments) indicate the class separations in the figures. Balanced data results (SMOTE versions) use the same colors and sizes for segments and classes. Class separations with the related segments are stated on the right sides of the unbalanced results in Figure 9a, Figure 10a, Figure 11a, and Figure 12a.

It can be easily seen from those lines that data accuracy increases gradually as the class separations diverge from the midpoint (5) of the VAD space for most of the classifications. For instance, the 3–7 class separation has higher accuracy values than the 4–6 separation, and the 4–6 is bigger than the 5 separation for most classifications.

**Figure 15:**
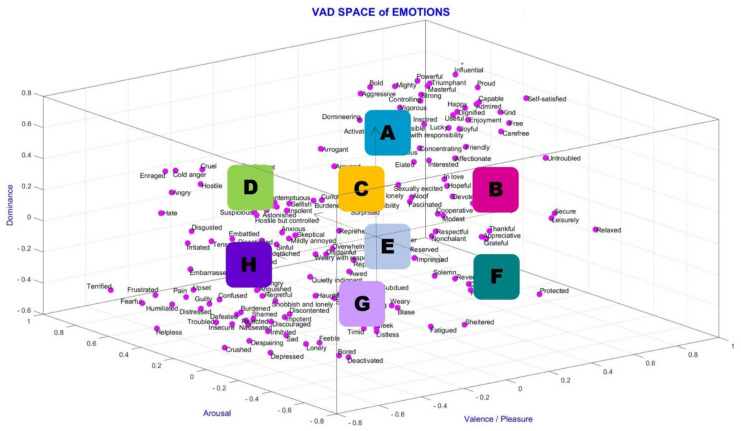
VAD space of emotions with class placements (VAD space is drawn by using the data in [14] and taking the pleasure plane as valence). The class placements are marked on the figure.

In this study, classes are set by considering the high and low values of the valence, arousal, and dominance scores. Class placements on VAD space are shown in Figure 15, in which the emotions are placed in the eight regions. To simplify understanding, the valence dimension is considered high and low (not as positive or negative), just like this study’s other dimensions (valence and dominance).

Bălan et al. [3] considered six basic emotions in their study: anger: low V, high A, D [6; 7]; joy: high V, high A, D [6; 7]; surprise: high V, high A, D [4; 5]; disgust: low V, high A, D [5; 6]; fear: low V, high A, D [3; 4]; and sadness: low V, low A, D [3; 4] for a 1–9 scale, as low indicates [1; 5] and high indicates [5; 9] intervals.

Looking mainly at Figure 15 and considering the 3D emotion space figures in [50] and [51] and the definitions in [3], we generally considered and inferred the eight three-dimensional emotion regions as:

A: Happy, joyful, useful, powerful, influential, friendly, excited, and inspired
B: Relaxed, leisurely, untroubled, and satisfied
C: Disdainful and unconcerned
D: Angry, hostile, cruel, insolent, hate, and disgusted
E: Impressed, surprised, thankful, and curious
F: Protected, humble, and reverent
G: Sad, depressed, bored, lonely, feeble, discouraged, and discontented
H: Fearful, frustrated, helpless, pain, humiliated, embarrassed, guilty, and confused

According to this information, we can state four cross-comparisons of VAD space in a briefer way as:

A&G (Happy, joyful, excited, and powerful and sad, depressed, and feeble)
B&H (Relaxed, leisurely, and satisfied and fear, pain, and guilt)
D&F (Angry, hate, and disgusted and protected, humble, and reverent)
C&E (Disdainful and unconcerned and impressed, surprised, and thankful).

Glancing at this inferred information, for the emotion regions with high valence values, which are A and B (with high dominance values) and E and F (with low dominance values), generally, we see positive emotions. On the other hand, for emotion regions with low valence values, which are G and H (with low dominance values) and C and D (with high dominance values), generally, we see negative emotions in accordance with the literature.

The best CCI, MCC values, and F1 scores (%) among Segments III, IV, and V for the cross-comparisons of VAD space are given in Table 3 for the unbalanced data and in Table 4 for the balanced data. It can be easily seen that Segment V gave mostly the best percentages for all CCI, MCC values, and F1 scores among Segments III, IV, and V. Besides these results, the RSS classifier gave the best percentages by a considerable amount with the unbalanced data, while the OF classifier gave similar results with the balanced data among the mentioned segments.

The best accuracies of the cross-comparisons for each class separation level can be easily seen in both tables. The SMOTE method considerably increases the percentages of the performance metrics, which can be seen in Table 4. As the class separations move away from the middle point (5), the accuracy values increase in general, which can also be seen in both of the tables.

For the A&G classes, which compare happy, joyful, excited, and powerful and sad, depressed, and feeble emotions, for the class separation levels 5 and 4–6, accuracy rates of 88.62% and 91.45% (CCI) are obtained, respectively, with the RF classifier and Seg V. For the 3–7 class separation level, an accuracy rate of 95.58% (CCI) is obtained for the SMO classifier according to the data-balanced results.

For the B&H classes, which compare relaxed, leisurely, and satisfied and fear, pain, and guilt emotions, for the class separation levels 5 and 4–6, accuracy rates of 82.60% and 93.37% (CCI) are obtained, respectively, with the OF classifier and Seg V. For the 3–7 class separation level, an accuracy rate of 100% (CCI) is obtained with both RF and OF classifiers according to the data-balanced results.

For the D&F classes, which compare angry, hate, and disgusted and protected, humble, and reverent emotions, for the class separation level 5, an accuracy rate of 89.94% (CCI) is obtained with the OF classifier and Seg V, and for the class separation level 4–6, an accuracy rate of 94.76% (CCI) is obtained for all IBk, SMO (Seg IV), and RC (Seg III and V) classifiers. For the class separation level 3–7, an accuracy rate of 95.05% (CCI) is obtained for the SMO classifier and Seg V according to the data-balanced results.

For the C&E classes, which compare disdainful and unconcerned and impressed, surprised, and thankful emotions, for the class separation level 5, an accuracy rate of 90.31% (CCI) is obtained with the OF classifier and Seg V, and for the class separation level 4–6, an accuracy rate of 97.92% (CCI) is obtained for the RC classifier and Seg III. For the class separation level 4.5–5.5, an accuracy rate of 97.92% (CCI) is obtained for the IBk classifier and Seg III according to the data-balanced results.

The best accuracy result in this study is for the C&E comparison for the unbalanced data (98.94% CCI with IBk classifier and Seg III for the 4.5–5.5 class separation). For the C&E comparison and the class separation level 4–6, an accuracy rate of 96.97% (CCI) is obtained with the FURIA classifier and Seg IV according to the unbalanced data results. For the B&H comparison and the class separation level 3–7, an accuracy rate of 96.03% (CCI) is obtained with the RC classifier and Seg V according to the unbalanced data results.

CCI, MCC values, and F1 scores (%) for the comparisons of VAD space for Segment V and the 4–6 classification are given in Figure 13 and Figure 14 (data-balanced version) for Random Forest, SMO, IBk, FURIA, and Random Committee classifiers. CCI (%) values generally gave close results to F1 scores (%); however, MCC values (%) gave relatively lower results, which can be seen in Figure 13 and Figure 14 and in Table 3 and Table 4.

Studies in the literature on emotion evaluation show that emotional states can be evaluated with discrete or dimensional emotion models. Dimensional models include Valence Arousal (VA) or Valence Arousal and Dominance (VAD) spaces. A comparison of our study with some studies that have a dominance dimension is given in Table 5. We can see that most of the studies in the literature use only VA due to the ease of differentiation; however, very few studies use VAD space like ours. Similarly, glancing over the studies in the literature for the segmentation of emotion analysis with handcrafted features, we also see that VA space is used. Considering those two issues, we especially wanted to focus on VAD space to compare and evaluate the emotional states and segmentations of EEG signals and segment comparisons in terms of emotion analysis. Based on this, we think this study will contribute to the literature.

In the literature, studies generally do not use so many comparison factors. In this context, the present study is critical because it offers an overall comprehensive and analytical approach with several different steps and complete comparisons applied for various factors for comparisons, such as binary cross-comparisons and evaluations of eight emotional states; comparisons of selected segments; comparisons of class separation levels (5, 4–6, and 3–7); and comparisons of unbalanced and balanced data with the SMOTE method. Giving detailed comparison results with the same or similar conditions is crucial. For instance, for these segmentation studies and binary comparisons of eight emotional states as well as other comparison factors, all the conditions are the same, including the same dataset, the same preprocessed EEG signal, the same channels, the same feature sets, the same class separations, the same classifiers, etc. As such, the results could be considered to show the differences in a more convenient and reliable way.

In our study, we have obtained high-accuracy results that can be seen directly in the spider web diagrams in the second part of this study and in Table 3 and Table 4. The best accuracy result in this study for unbalanced data is for the C&E comparison (Seg III and 4.5–5.5 class separation), for which an accuracy rate of 98.94% CCI (F1: 98.9% and MCC: 97.9%) is obtained with the IBk classifier, which is greater than the compared studies from the literature that have a dominance dimension besides valence and arousal, as shown in Table 5.

The overall analysis results conducted with VAD space show that accuracy values obtained from C&E comparisons were generally higher than all the other cross-comparisons for unbalanced data. From all the results in Figure 6 and Figure 7 (data-balanced version) in the first part of this study, accuracy rates generally seem to decrease gradually in the order of C&E, D&F, B&H, and A&G comparisons, respectively, for 4–6 class separations.

Besides these results, if we look at all the results and Figure 9a, Figure 10a, Figure 11a, and Figure 12a in the second part of this study, the accuracy rates generally seem to gradually decrease in the order of C&E, B&H, D&F, and A&G comparisons, respectively. Here, C&E and B&H seem similar at a glance; however, we should remember that C&E comparisons do not include the 3–7 class separation. As such, a middle-sized class separation (4–6) in B&H and other comparisons correspond to a thick-sized class separation (4–6) in C&E, as seen in Figure 12a,b. Data-balanced versions mostly seem to outperform the unbalanced versions in this study, as seen in the figures of the second part of this study.

A possible limitation of this study is that class-level separations could decrease the balance among data classes. Oversampling and down-sampling methods could not give sufficient repairments of the proposed methods. For instance, if the data have more unbalanced classes, this would require a more significant percentage of oversampling of the minority class or a more substantial percentage of down-sampling of the majority class.

We plan to apply the analysis to some other databases in future work. We plan to compare the emotions as changing parameters and emotions in VA/VAD spaces. We are also planning to try some other classifiers. In future applications, the obtained methods and information could be used with modifications for clinical research on emotion analysis. For example, this might involve evaluating a group’s emotions that appear due to stimuli applied according to the experimental design. Another example is BCI technologies, which have important contributions to medical applications. For example, BCI devices, such as wheelchairs, robotic arms, and drones, can easily turn left and right and move forward based on left and right hand motor imagery. Neural activities related to inhibition (P300) can be applied as a stop command [52]. Chikara et al. [52] developed a model for identifying the neural activities of human inhibition using a phase-locking value method. Their results imply that these activities can be used as a stop command in BCI technologies and to identify the symptoms of attention deficit hyperactivity disorder patients in clinical research. Furthermore, as future applications, the classification and comparison of emotions could be used in many areas, including smart health technologies, psychology, robot technologies, simulation systems, game technologies, smart homes, driver systems, etc.

## 5. Conclusions

A comprehensive analytical approach is implemented with two sub-studies: first, a segmentation study, and second, an emotion comparison and evaluation in addition to comparisons of selected segments in a more detailed way. This was performed in addition to comparing the effect of class separation levels and classification accuracies for both unbalanced and balanced feature sets. The results obtained from the second part of this study are compared, evaluated, and commented on with this information. Eight emotional states are considered and inferred for VAD space.

This study is critical because it compares segmentation, class separation levels, and the data balancing effect with SMOTE. The segmentation effect is significant because it is conducted with 3D VAD space with handcrafted features, including the dominance dimension in the evaluation, while also considering the impact of each of the three dimensions together. In conclusion, it is emphasized that emotion evaluation can be conducted with EEG signals successfully. It is believed that because facial expressions or speech could be misleading in some conditions that could change from person to person, a physiological signal obtained from the brain may give closer results to the emerging emotion. Consequently, the results emphasize that emotional states occur due to stimuli, such as music videos, which can change the physiological signals and can be predicted by analyzing the EEG data.

## Figures and Tables

**Table 1:** Class separation levels.

Class Separation Level	High (H) ^1^	Low (L) ^1^
5	≥5	<5
4.5–5.5 ^2^	≥5.5	≤4.5
4–6	≥6	≤4
3–7	≥7	≤3

^1^ For each 3 dimensions (valence, arousal, and dominance) ^2^ 4.5–5.5 class separation level has only been used for C&E emotion comparisons.

**Table 2:** Segmentation of EEG signals.

Segment Name	Windowing (Second)	Segment/Piece
*N*	None	1
Seg I	30	2
Seg II	20	3
Seg III	15	4
Seg IV	10	6
Seg V	5	12
Seg VI	3	20
Seg VII	2	30
Seg VIII	1	60

**Table 3:** Best CCI, MCC values, and F1 Scores (%) among Segments III, IV, and V for the cross-comparisons of VAD space.

Sep. Level	Class	CCI (%)			F1 Score (%)			MCC (%)		
		Value	Classifier *	Segment	Value	Classifier	Segment	Value	Classifier	Segment
(5)	A&G	81.71	RSS	V	80.5	RSS	V	54.9	RSS	V
	B&H	81.98	RSS	V	81.9	RSS	V	63.8	RSS	V
	D&F	85.68	RF	V	85.5	RF	V	69.2	RF	V
	C&E	86.84	RSS	V	86.8	RSS	V	72.9	RSS	V
(4–6)	A&G	85.13	RSS	V	84.3	RSS	V	62.3	RSS	V
	B&H	91.01	OF	V	90.9	OF	V	80.7	OF	V
	D&F	92.35	RF	V	92.3	RF	V	82.9	RF	V
	C&E	96.97	FURIA	IV	97.0	FURIA	IV	93.9	FURIA	IV
(3–7)	A&G	90.08	RSS	V	89.6	RSS	V	72.2	RSS	V
	B&H	96.03	RC	V	95.9	RC	V	85.2	RC	V
	D&F	91.32	RSS	V	91.4	RSS	V	81.3	RSS	V
(4.5–5.5)	C&E	98.94	IBk	III	98.9	IBk	III	97.9	IBk	III

* FURIA: Fuzzy Unordered Rule Induction Algorithm; IBk: Instance-Based Learning (it is a K-Nearest Neighbor classifier); OF: Optimized Forest; RC: Random Committee; RF: Random Forest; RSS: Random Subspace.

**Table 4:** Best CCI, MCC values, and F1 scores (%) among Segments III, IV, and V for the cross-comparisons of VAD space (balanced data with SMOTE).

Sep. Level	Class	CCI (%)			F1 Score (%)			MCC (%)		
		Value	Classifier *	Segment	Value	Classifier	Segment	Value	Classifier	Segment
(5)	A&G	88.62	RF	V	88.6	RF	IV, V	77.4	RF	V
	B&H	82.60	OF	V	82.6	OF	V	65.2	OF	V
	D&F	89.94	OF	V	89.9	RF, OF	V	80.2	OF	V
	C&E	90.31	OF	V	90.3	OF	V	80.7	OF	V
(4–6)	A&G	91.45	RF	V	91.4	RF	V	83.1	RF	V
	B&H	93.37	OF	V	93.4	OF	V	90.8	FURIA	III
	D&F	94.76	IBk, RC, SMO	III, IV, V	94.8	IBk, RC, SMO	III, IV, V	90.0	IBk	IV
	C&E	97.92	RC	III	97.9	RC	III	95.9	RC	III
(3–7)	A&G	95.58	SMO	IV	95.6	SMO	IV	91.4	SMO	IV
	B&H	100.00	RF, OF	III	100.0	RF, OF	III	100.0	RF, OF	III
	D&F	95.05	SMO	V	95.0	SMO	V	90.3	SMO	V
(4.5–5.5)	C&E	97.92	IBk	III	97.9	IBk	III	95.9	IBk	III

* FURIA: Fuzzy Unordered Rule Induction Algorithm; IBk: Instance-Based Learning (it is a K-Nearest Neighbor classifier); OF: Optimized Forest; RC: Random Committee; RF: Random Forest; RSS: Random Subspace; SMO: Sequential Minimum Optimization.

**Table 5:** Comparison with some studies that use the dominance dimension.

Study	Class	Accuracy ^1^
[12]	3-class classification (Low, medium, and high)	63.47 (V)
		69.62 (A)
		63.57 (D)
[15]	2 classes	73.43% (V)
		72.65% (A)
		69.3% (D)
[16]	2D and 3D emotion models	
	4 emotional states (VA model)	Best accuracy of 79.1%.
	8 emotional states (VAD model)	Highest accuracy of 93%
Our Study	Binary classification for cross-comparisons of 8 emotional states from VAD space (A&G; B&H; D&F; C&E) for unbalanced data	Best accuracy of 98.94% CCI (F1: 98.9% and MCC: 97.9%) for C&E comparison (Seg III and 4.5–5.5 class separation)
		Please see Table 3 for other results.
	for balanced data with SMOTE	Best accuracy of 100% CCI, F1 and MCC for B&H comparison (Seg III and 3–7 class separation)
		Please see Table 4 for other results.

^1^ Valence: V; Arousal: A; Dominance: D.

## Data Availability

The DEAP dataset used in this study is available at: http://www.eecs.qmul.ac.uk/mmv/datasets/deap/ and can be accessed upon approval (accessed on 14 September 2021).
